# Different in the dark: The effect of habitat characteristics on community composition and beta diversity in bromeliad microfauna

**DOI:** 10.1371/journal.pone.0191426

**Published:** 2018-02-05

**Authors:** Annika Busse, Pablo A. P. Antiqueira, Alexandre S. Neutzling, Anna M. Wolf, Gustavo Q. Romero, Jana S. Petermann

**Affiliations:** 1 Department of Biosciences, University of Salzburg, Salzburg, Austria; 2 Berlin-Brandenburg Institute of Advanced Biodiversity Research (BBIB), Berlin, Germany; 3 Graduate Program in Ecology, Institute of Biology, University of Campinas (UNICAMP), Campinas-SP, Brazil; 4 Multitrophic Interactions and Biodiversity Lab, Department of Animal Biology, Institute of Biology, University of Campinas (UNICAMP), Campinas-SP, Brazil; 5 Institute of Biology, Freie Universität Berlin, Berlin, Germany; Universidade Federal de Goias, BRAZIL

## Abstract

The mechanisms which structure communities have been the focus of a large body of research. Here, we address the question if habitat characteristics describing habitat quality may drive changes in community composition and beta diversity of bromeliad-inhabiting microfauna. In our system, changes in canopy cover along an environmental gradient may affect resource availability, disturbance in form of daily water temperature fluctuations and predation, and thus may lead to changes in community structure of bromeliad microfauna through differences in habitat quality along this gradient. Indeed, we observed distinct changes in microfauna community composition along the environmental gradient explained by changes in the extent of daily water temperature fluctuations. We found beta diversity to be higher under low habitat quality (low canopy cover) than under high habitat quality (high canopy cover), which could potentially be explained by a higher relative importance of stochastic processes under low habitat quality. We also partitioned beta diversity into turnover and nestedness components and we found a nested pattern of beta diversity along the environmental gradient, with communities from the lower-quality habitat being nested subsets of communities from the higher-quality habitat. However, this pattern resulted from an increase in microfauna alpha diversity with an increase in habitat quality. By providing insights into microfauna-environment relationships our results contribute to the mechanistic understanding of community dynamics in small freshwater bodies. Here, we highlight the importance of habitat characteristics representing habitat quality in structuring communities, and suggest that this information may help to improve conservation practices of small freshwater ecosystems.

## Introduction

Detailed information about an ecosystem and its structuring processes is crucial for development of effective and sustainable conservation strategies for biodiversity maintenance [1]. Especially freshwater ecosystems, which hold a high proportion of species, are experiencing unprecedented declines in biodiversity [2] and are in need of suitable conservation measures [3]. Earlier studies have shown that parameters like disturbances [4], toxic substances [5] and spatial connectivity [6] can potentially affect freshwater community composition. Also, several likely drivers of species diversity, e.g. elevation [7] and acidity [8], and their abundances, e.g. resource availability [9] and predation [10], have been identified. More recently, it has been noted that for effective conservation the distribution of biodiversity in space has to be taken into account [11]. Therefore, the drivers of differences in community composition along spatial or environmental gradients have come into the focus of ecological research [12–15].

In general, community similarity is assumed to decrease with larger environmental or spatial distances between communities [16]. The magnitude of differences in community composition is commonly measured as beta diversity [16–19]. For example, high beta diversity indicates large differences in composition among local communities within a habitat. Previous research demonstrated that beta diversity can depend on a number of different processes. For example, it can be reduced by strong competitive exclusion [20], i.e. conditions where deterministic processes dominate. Beta diversity can also be increased in case of dispersal limitation [21, 22] or when high rates of random extinction and immigration events led to distinct demographic stochasticity [20, 23]. Thus, changes in beta diversity seem to be observed when different types of community-structuring processes change in their relative importance, with a high relative importance of deterministic processes potentially leading to smaller beta diversity and vice versa [24, 25].

The current knowledge of the conditions under which deterministic versus stochastic processes dominate in their relative importance and affect beta diversity is still ambiguous. For example, harsh environmental conditions, i.e. low habitat quality, may, on the one hand, reduce beta diversity due to strong environmental filtering [26] or, on the other hand, increase beta diversity due to dispersal limitation [27]. It has been long known that environmental harshness (or habitat quality) is a key factor in driving community composition by affecting assembly and maintenance processes [26, 28]. However, the contradictory evidence described above indicates that the ecological mechanisms governing the natural patterns of beta diversity remain to be explained. Here, we provide further insights into beta diversity disparities by conducting the first study on drivers of microfauna beta diversity in small freshwater bodies along a habitat quality gradient.

We use freshwater microhabitats found in tank bromeliads. These natural microcosms constitute useful model systems for testing various questions in ecology because they are relatively small and easily sampled micro-ecosystems with clear boundaries that can be measured in their entirety [7, 29, 30]. Bromeliad-inhabiting communities are per definition metacommunities which “are linked by dispersal of multiple potentially interacting species” [31] and are therefore especially suitable to address research questions related to patterns of community composition along spatial or environmental gradients. Moreover, bromeliads can occur in high densities [32, 33] which allows for many replicates under comparable environmental conditions. The pools between the leaf axils of water-collecting tank bromeliads are typically colonized by a variety of aquatic organisms. They comprise many different taxa of protists, small metazoans and insect larvae which form a food web based on decomposing leaf litter that falls from the canopy in the tank [34, 35].

We investigate bromeliads along a canopy cover gradient in restinga forest in Brazil. Strong but variable impacts of canopy cover on bromeliad-inhabiting microfauna and invertebrate communities have been observed in former studies [36–38]. With our study we aim to identify the canopy-cover related factors that affect bromeliad-inhabiting microfauna communities. The more open sites have lower densities of trees and thus, the bromeliads are exposed to direct sunlight. The more forested sites have a higher density of trees and thus, constitute a more shaded habitat for the bromeliads, thereby potentially providing higher resource amounts for the bromeliad microfauna in terms of greater leaf litter input [39, 40]. The addition of these resources has experimentally been shown to favour flagellates and ciliates over algae and amoebae and thus may lead to a shift in community composition [34].

Another likely difference between the bromeliads along the canopy cover gradient, which results from the degree of exposedness to the sun, is the daily variation in water temperature. Daily fluctuations in water temperature are expected to decrease with increasing canopy cover and could potentially affect microfauna richness [36]. It has been shown that temperature fluctuations in general and their strength in particular can affect species coexistence and thus diversity [41, 42]. As a further difference between communities of different canopy cover, the abundance of protist-feeding mosquito larvae is known to be much higher in sun-exposed bromeliads than in shaded bromeliads (*P*.*A*.*P*. *Antiqueira & G*.*Q*. *Romero unpublished data*). This predation by unselective filter-feeders might also influence community composition, e.g. through predator-mediated coexistence [43].

**Fig 1:**
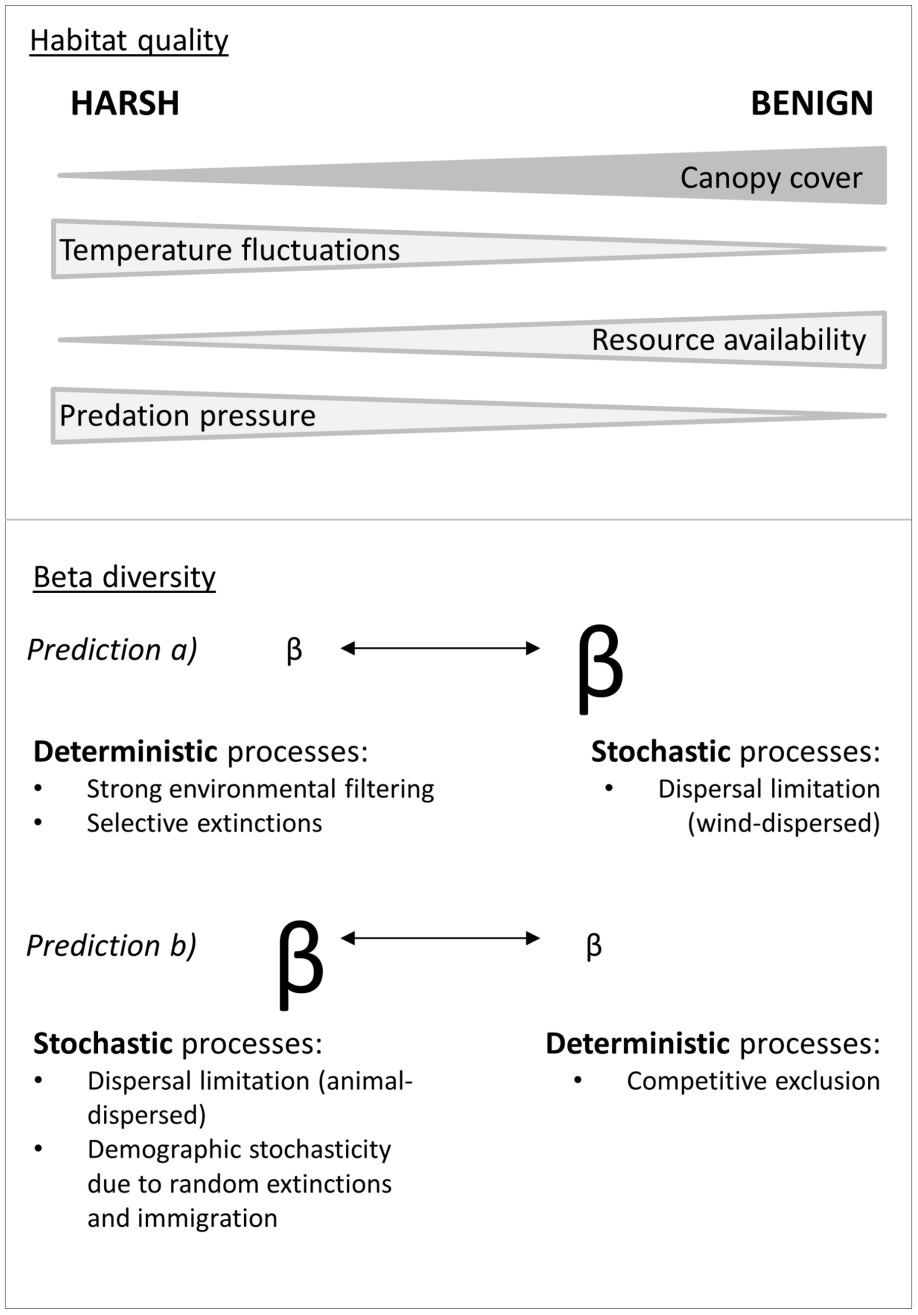
Contrasting predictions concerning the differences in beta diversity of bromeliad-inhabiting microfauna along a habitat quality gradient. At the top, the direction of canopy cover and canopy cover-related factors is given along a habitat-quality gradient. At the bottom, hypothesized beta diversity differences, related processes and mechanisms are shown. According to our first prediction a) of our hypothesis 2), harsh environmental conditions (e.g. higher daily temperature fluctuations and low nutrient availability) result in low beta diversity due to selective extinctions driven by strong environmental filtering. In the more benign but also more forested habitat, trees and dense shrub vegetation may have an effect on microfauna community composition if dispersal of microfauna (or their cysts) is mainly wind-driven. According to the second prediction b) of our hypothesis 2), dispersal limitation is stronger in the harsher habitat assuming that microfauna is primarily dispersed by animals (instead of wind) and that these are less active in the harsher, more exposed area. Furthermore, random extinction (e.g. through higher predation pressure by unselective filter-feeders) and immigration events are probably contributing to higher beta diversity in the harsh habitat while competitive exclusion might lower beta diversity in the benign habitat.

In short, bromeliad microcosms may vary along the canopy cover gradient in three major aspects (Fig 1). First, an increase in canopy cover leads to an increase in resource availability. Second, an increase in canopy cover leads to a decrease in solar radiation and thus to less pronounced daily temperature fluctuations. Third, an increase in canopy cover is accompanied by a decrease in predation pressure. Thus, an increase in canopy cover is accompanied by a number of favourable circumstances (e.g. sufficient resource availability, more constant environmental conditions and less predation) which result in less environmental stress and more advantages for the bromeliad-inhabiting microfauna. Hence, the canopy cover gradient likely constitutes a gradient of habitat quality for bromeliad-inhabiting microfauna communities with harsher conditions (lower habitat quality) in more sun-exposed sites and more benign conditions (higher habitat quality) in more forested sites. We use this habitat-quality gradient to study how habitat characteristics may affect community structure, specifically alpha diversity, community composition, beta diversity (Fig 1) and the beta-diversity components nestedness and turnover.

We hypothesize that:

1. Community composition of bromeliad-inhabiting microfauna decreases in similarity with increasing environmental distance along the canopy cover gradient. These differences in community composition are driven by environmental variables that are directly or indirectly related to canopy cover.
2. Beta diversity of bromeliad microfauna changes along the canopy cover gradient due to differences in habitat quality. Whether beta diversity increases or decreases with increasing habitat quality, i.e. with increasing canopy cover, may depend on the relative importance of different types of coexistence processes (i.e. stochastic versus deterministic processes). Thus, we formulate two contrasting expectations (see also Fig 1).
  - Beta diversity increases with increasing habitat quality, suggesting strong environmental filtering in the harsher environment and higher dispersal limitation in the more benign environment.
  - Beta diversity decreases with increasing habitat quality, suggesting higher demographic stochasticity and/or dispersal limitation in the harsher environment and stronger competitive exclusion in the more benign environment.

## Methods

### Study site and system

Samples were taken on Cardoso Island at the south coast of Sao Paulo State, Brazil (25° 03’S, 48°53’W), in September 2013 at the beginning of the wet season. Cardoso island is characterized by mean annual temperatures between 20 and 22°C and mean annual rainfall of 2250 mm [44]. Relative humidity is over 66% in spring (= sampling season of our study) in restinga habitats [45]. Our study was carried out in the northern part of the island within an area of 4.5 km extension. The study site was situated in restinga rainforest, a type of Atlantic rainforest on coastal dunes [46]. On Cardoso Island, restinga rainforest show different vegetation and abiotic conditions along a canopy cover gradient. Less forested restinga (i.e. more sun-exposed habitats for bromeliads and their microfauna communities) and more forested restinga (i.e. more shaded habitats for bromeliads and their microfauna communities). In the less forested habitat, shrub vegetation (maximum 4 m high) is distributed in patches containing lianas with sun-exposed areas between these patches. In the more forested habitat trees range from 6 to 8 m height and may form a relatively continuous canopy cover. Bromeliad density was higher in the more forested restinga (*personal observation*).

Microfauna communities were sampled from water-filled leaf axils of these bromeliad plants. Plant-held waters are commonly referred to as phytotelmata, of which bromeliads constitute only one possible type [32]. Tank bromeliads occur almost exclusively in the Neotropics growing on ground level or as epiphytes on branches or trunks. Their funnel-shaped leaf morphology with numerous leaf compartments captures water from above (i.e. rainwater or stem flow) and falling leaf litter from the canopy. Aquatic decomposers such as protozoa and nematodes break down the leaf litter and make the nutrients available for other organisms in the leaf compartment pool and the bromeliad plant. Furthermore, decomposers are prey to various predators within the tank, including larger protozoa, rotifers and insect larvae. This study focuses on microfauna communities including organisms of the size class 5–200 μm such as protozoa (including flagellates, ciliates and amoebae) and rotifers. Bromeliad tanks can occur in high densities in the tropical rainforest holding up to 50,000 L water/hectare [47]. As such, bromeliad tanks constitute valuable freshwater habitats in the tropics, and may provide important ecological functions, amongst others by being the main breeding ground for semiaquatic insects. Interspecific interactions are not confined to the aquatic bromeliad tank but include the surrounding terrestrial environment because bromeliads provide drinking water reservoirs and preying grounds for many species [48–50]. Last but not least, bromeliad microcosms can contain endemic species which are highly adapted to the phytotelm environment thus enhancing species diversity by providing ecological niches [51, 52]. Apart from their ecological importance, bromeliads provide valuable model systems for community research and questions related to the metacommunity concept.

### Experimental design

In a stratified random sampling design including four different sites with more sun-exposed or more forested restinga rainforest we selected 78 tank bromeliads of the species *Quesnelia arvensis* Mez. (Bromeliaceae) growing on ground level. We collected similar-sized bromeliads (total water volume, mean ± SE: 1386 ± 106 mL) to reduce the effect of habitat size on the studied communities.

### Sampling

Portable digital thermometer data loggers (Thermochron^®^ iButton^®^ device—DS1921G) were added to all bromeliads prior to sampling to register the water temperature variation of each bromeliad. From these recorded temperature data three different temperature measurements were calculated: average water temperature, maximum water temperature and coefficient of variation of water temperature (calculated for a time frame of 23 hours). The three variables were strongly correlated (Pearson’s correlation: p ≤ 0.001 for all correlation pairs). To avoid multicollinearity in our analyses, we chose one of the three variables for further analyses: the coefficient of variation, which we considered to be the most representative temperature measurement. The canopy cover was determined for each bromeliad by analysing canopy photos with the program ImageJ [53]. Furthermore, during sampling a set of parameters, representing potentially important abiotic and biotic environmental drivers of microfauna community composition, were measured for each bromeliad. First, the number of water-filled bromeliad leaf compartments was counted and bromeliad diameter [cm] and vertical height [cm] were measured. Dissolved oxygen concentration [%] and pH were measured in the field using a multiparameter handheld meter (cyberscan PD 650, Oaklon^®^). Furthermore, a water sample was collected to analyze turbidity [NTU = nephelometric turbidity unit], chlorophyll a concentration [μg/L], carbon dissolved organic matter (CDOM) [ppb] and ammonium concentration [μM] using a handheld fluorometer (AquaFluor^®^). Afterwards, to survey the microfauna, a 1 mL water sample per bromeliad was taken from a leaf compartment halfway between the central and outermost leaf compartments and fixed with Lugol’s solution. Microfauna were counted as morphotypes for 50 μL of each sample using light microscopy (400 x magnifications). Moreover, the abundance of mosquito larvae per bromeliad was counted in a sample of 17-100mL water (depending on the available volume; mean +/- SE: 80 +/- 2 mL) and the number of mosquitos/100mL was calculated. Total water volume was determined for each bromeliad by extracting all the water.

The study did not involve endangered or protected species. Sampling was carried out under permit 23689–1 issued by Instituto Chico Mendes de Conservação da Biodiversidade.

### Statistical analysis

#### Pairwise dissimilarities and singleton removal

To detect the dissimilarity index best suited to describe our abundance-based data, we performed a preliminary rank index analysis [54] using the R package *vegan* [55]. The Bray-Curtis dissimilarity index was identified as the most suitable and was used to obtain an abundance-based dissimilarity matrix for further analyses.

We tested the effect of singleton removal by comparing non-metric multidimensional scaling ordinations (NMDS) with 20 random starts for data sets with and without singletons. Three different definitions of “singletons” were tested according to Poos and Jackson [56]: singletons are defined as species that occur i) in only one site (in our case 3 morphospecies were removed), ii) in less than 5% of sites (11 morphospecies were removed) and iii) in less than 10% of sites (17 morphospecies were removed). Procrustes correlation analysis (999 permutations) was used to identify the significance of the congruence between the ordinations with singleton removal and the ordination on the complete data set. None of the three singleton removal strategies showed a significant difference for the community composition (Procrustes correlation coefficient > 0.95, p-value < 0.001 for all three comparisons). Therefore, no singletons were removed prior to statistical analysis.

#### Community composition and environment

The environmental variables that we measured were tested for multicollinearity. Decisions to remove redundant predictors were based on a combination of correlation coefficients, cluster analysis and biological relevance. After reduction of redundant environmental variables all statistics were done using the following seven of the originally thirteen environmental variables: canopy cover, number of leaf compartments, coefficient of variation of water temperature, pH, turbidity, dissolved oxygen concentration and mosquito larvae abundance.

To determine if community composition changes along the canopy cover gradient we calculated a distance decay plot. It tests for pairwise dissimilarities along an environmental gradient, whereby according to our hypothesis 1) an increase in the difference of canopy cover was expected to result in increasing Bray-Curtis dissimilarity values due to increasing differences in environmental conditions. This relationship was tested with a multiple regression on distance matrices [57, 58] (using the MRM function in the R package *ecodist* [59]) that is based on permutation tests of significance (999 permutations).

To further investigate which of the canopy cover-related factors drive community composition in particular, we carried out a distance-based redundancy analysis (dbRDA), i.e. a constrained version of principal coordinates analysis (PCoA) [60] using measured environmental variables that are related to canopy cover changes (for details on the relationship between environment variables and canopy cover see S1 Fig). The statistical significances of the overall model and single model terms were tested with permutation tests.

#### Beta diversity

There are many possibilities to measure beta diversity and none of these is perfect [61]. To address the question if beta diversity depended on habitat quality we chose to use multivariate dispersion as a measure of beta diversity [62]. Because this analysis can only compare different levels of a categorical variable, we grouped the samples along our continuous canopy cover gradient into two groups (based on the median), the bromeliads in the more sun-exposed habitat, representing the low-quality (harsh) habitat, and the bromeliads in the more shaded habitat, representing the high-quality (benign) habitat. Each group contained 39 bromeliads of the 78 measured in total. For each bromeliad we calculated beta diversity as distance to group centroid based on a Bray-Curtis dissimilarity matrix by using the R function “betadisper” (R package *vegan* [55]). The calculated distances to group centroid of the two habitats were then compared using a linear model. To correct for a high influence of alpha diversity on patterns in beta diversity, a Raup-Crick null model [63] was applied and the resulting matrix was used to calculate differences in beta diversity.

When beta diversity is calculated using pairwise Sørensen dissimilarity, it can be partitioned into two components: turnover (replacement of species by other species in different sites) and nestedness (species loss or gain between sites) [64, 65]. To identify if differences in community composition were mainly due to species turnover or nestedness, beta diversity was partitioned using the R package *betapart* [64]. To assess whether the results for the turnover and nestedness components were greater than expected by chance, we used a null model with 10000 permutations. The null matrix was constrained by the “r1”-method [66] which maintains the row frequencies and uses column marginal frequencies as probabilities of selecting species. This method is based on z-scores with positive z values indicating a higher than expected contribution of the turnover or nestedness component.

To quantify the total degree of nestedness along the canopy cover gradient a NODF metric (nestedness measure based on overlap and decreasing fills) was applied [67, 68]. We used the nestedrank-function in the R package *bipartite* [69] to calculate the nestedness rank of communities along the canopy cover gradient. A high rank indicates a more nested community. We used a linear model to test for the effect of canopy cover on nestedness rank. As nestedness is a result of species loss we also tested if alpha diversity changes along the canopy cover gradient using a linear model. Resulting from this, we repeated the first linear model, testing for the effect of canopy cover on nestedness rank with alpha diversity as a co-variable fitted before canopy cover, to differentiate between the effect of alpha diversity and canopy cover on nestedness rank.

All statistical analyses were done in R version 3.0.2 [70] using the packages *vegan* [55], *betapart* [64], *bipartite* [69] and *ecodist* [59].

## Results

### Abundance and alpha diversity

**Fig 2:**
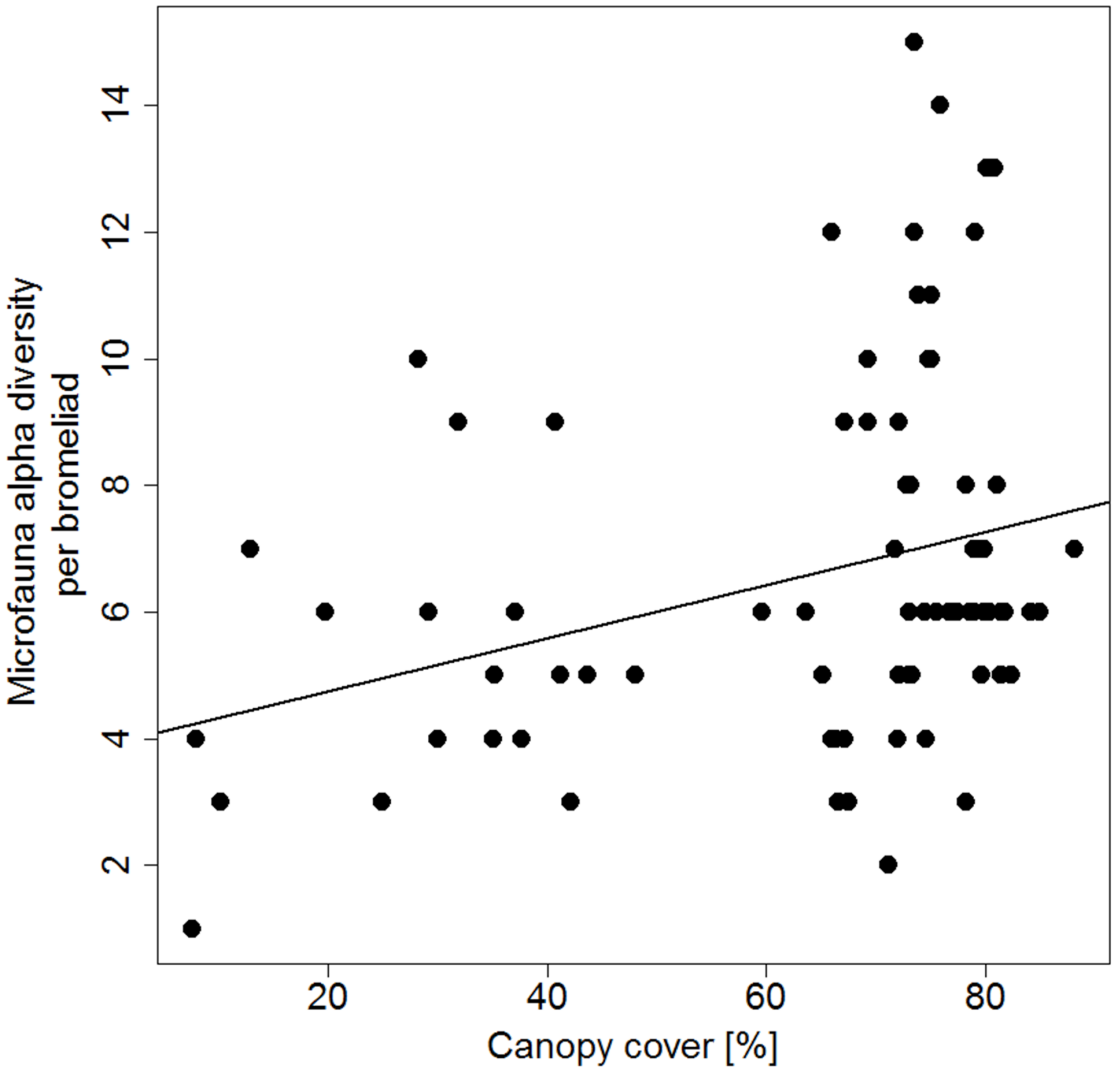
Mircofauna alpha diversity from bromeliads showing a positive linear relationship with canopy cover. Linear model: F_1,76_ = 7.8, p = 0.007. n = 78. Microfauna (including protozoa and small metazoa) were counted as morphotypes in 50 μL of Lugol-fixed water samples.

A total of 35 morphotypes of microfauna were identified from our samples, including flagellates (15 morphotypes), ciliates (9 morphotypes), amoebae (4 morphotypes) and rotifers (7 morphotypes). For a detailed description of morphotypes see S1 Table. On average, flagellates had the highest alpha diversity per bromeliad (2.5 morphotypes ± 0.2 SE/50 μl), followed by ciliates (2.0 morphotypes ± 0.1 SE/50 μl), rotifers (1.5 morphotypes ± 0.1 SE/50 μl) and amoebae (0.6 morphotypes ± 0.1 SE/50 μl). Flagellates also had the highest mean abundance (227 individuals ± 123 SE/50 μL) followed by ciliates (40 individuals ± 8 SE/50 μL), rotifers (6 individuals ± 1 SE/50 μL) and amoebae (5 individuals ± 2 SE/50 μL). Alpha diversity significantly increased with higher canopy cover (Linear model: F_1,76_ = 7.8, p = 0.007, Fig 2). Alpha diversity was not significantly related to any other explanatory variable measured in this study. Log-transformed total microfauna abundance was not related to canopy cover (Linear model: F_1,76_ = 2.1613, p = 0.1455).

### Community composition and environment

**Fig 3:**
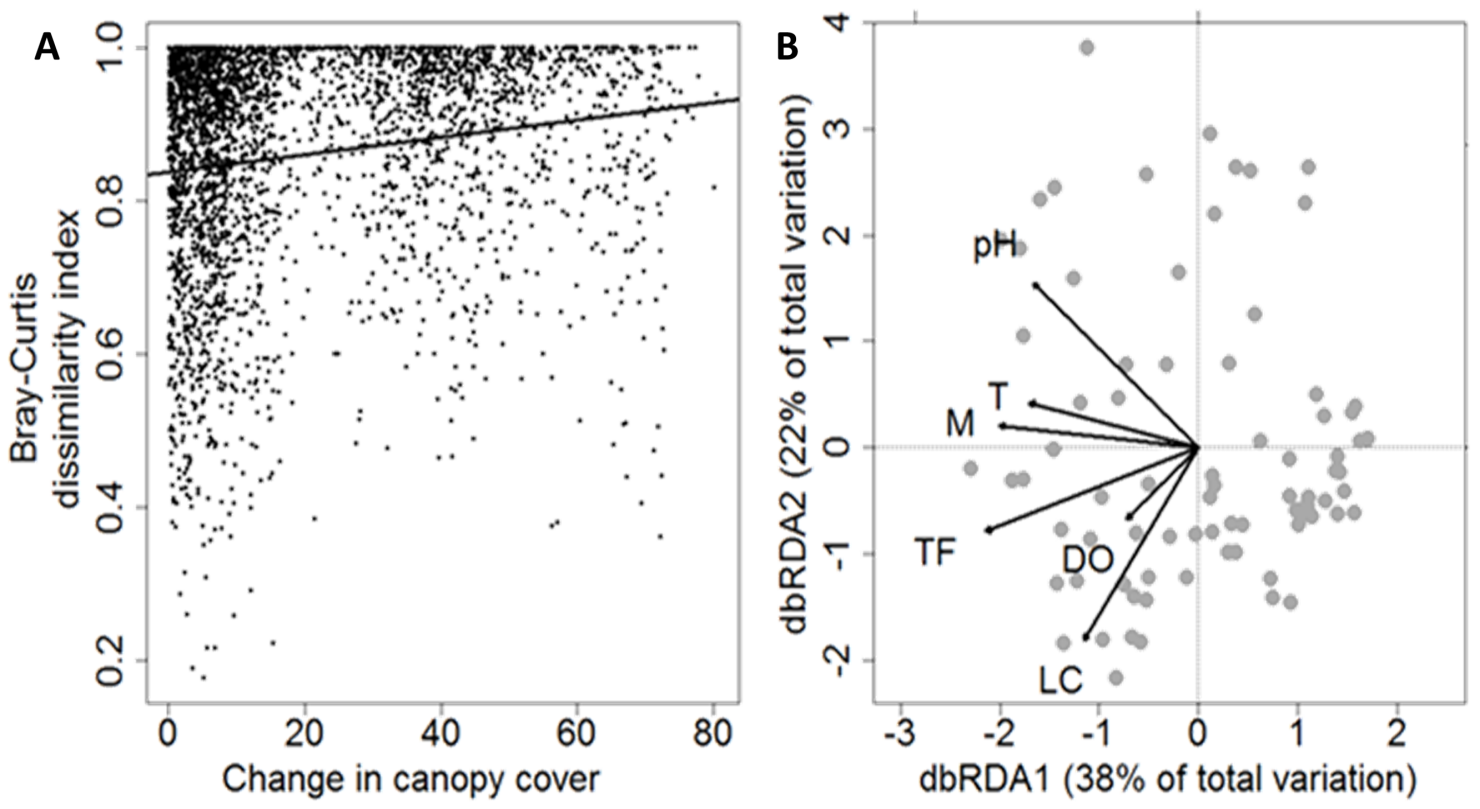
Drivers of bromeliad-inhabiting microfauna community composition. A: Distance-decay plot depicting the effect of change in canopy cover on community dissimilarity of bromeliad-inhabiting microfauna. The continuous line results from multiple regressions on distance matrices (MRM: R^2^ = 0.026, p = 0.001) to test for a linear relationship between change in canopy cover and community dissimilarity. B: Distance-based redundancy analysis of bromeliad-inhabiting microfauna communities illustrating the influence of canopy cover-related factors. pH—pH, T—turbidity [NTU], M—mosquito larvae [per 100 mL], TF—daily water temperature fluctuations measured as coefficient of variation, DO—dissolved oxygen concentration [%], LC—number of leaf compartments per bromeliad. n = 77. Daily fluctuations in water temperature explained the highest proportion of total variation (dbRDA1, Table 1).

We investigated if a linear relationship existed between distance in environmental conditions (i.e. canopy cover) and the dissimilarity of communities measured using the Bray-Curtis index. We found that with increasing differences in canopy cover bromeliad microfauna communities became more dissimilar (MRM: R^2^ = 0.026, p = 0.001, Fig 3A). So, community composition changed gradually along the canopy cover gradient. To identify the environmental factors through which changes in canopy cover affected community composition a distance-based RDA was applied (dbRDA model: F_70,6_ = 1.5, p < 0.001, Fig 3B). The model returned daily fluctuations in water temperature (represented by the coefficient of variation of water temperature), the number of leaf compartments and pH as significant drivers of community composition while the other environmental variables did not show significant effects on community composition (Table 1). We also used raw abundance of mosquito larvae and total water volume per bromeliad as co-variables in the analysis. However, this did not change the results.

**Table 1:** Results of a permutation test on the distance-based redundancy analysis of the effects of individual environmental variables on bromeliad-inhabiting microfauna communities. Variables with significant effects are highlighted in bold.

	Df	F	P
**Number of leaf compartments**	1	1.83	**0.023**
**Daily fluctuations in water temperature**	1	2.23	**0.003**
**pH**	1	1.85	**0.013**
Turbidity [NTU]	1	1.25	0.187
Dissolved oxygen concentration [%]	1	0.87	0.641
Mosquito larvae [per 100 mL]	1	1.13	0.282
Residuals	70		

Daily fluctuations in water temperature were measured as coefficient of variation. n = 77. Statistically significant effects are printed in bold. Df—degrees of freedom, F—F statistic indicating the variation between the group means, P—p value indicating the significance of the model parameters.

### Beta diversity

**Fig 4:**
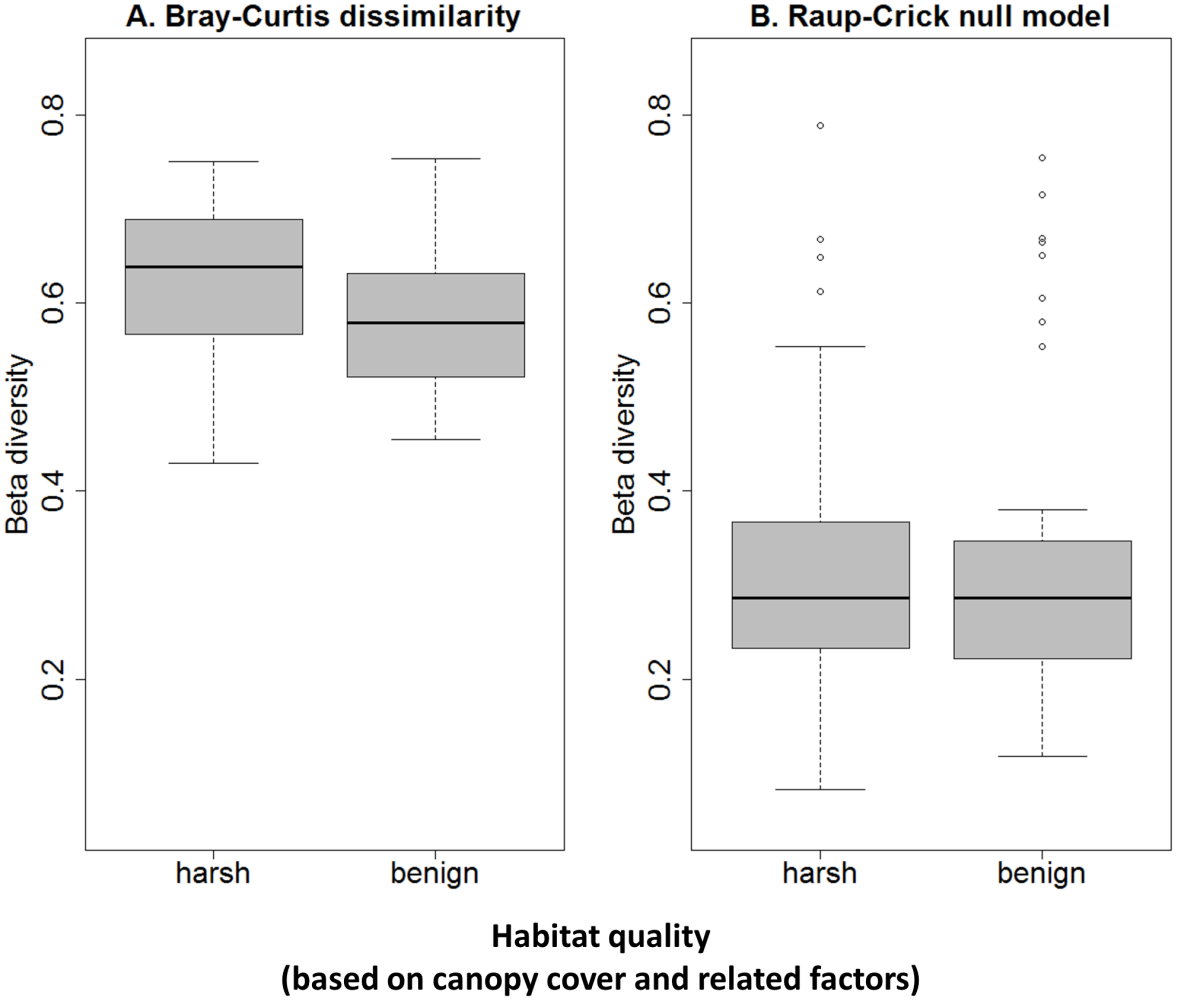
Beta diversity, measured as distance to group centroid, of bromeliad-inhabiting microfauna in two qualitatively distinct habitats. Habitat quality is defined based on canopy cover-related differences in predation pressure, temperature fluctuations and resource availability, which make the sun-exposed side an assumingly harsher habitat for microfauna than the shaded side. Beta diversity is significantly different when using a Bray-Curtis dissimilarity matrix (A, linear model, F_1,76_ = 10.1, p = 0.002). The significant difference is lost when using a Raup-Crick null model to correct for differences in alpha diversity (B, linear model, F_1,76_ = 0.1, p = 0.74). n = 78.

Beta diversity, measured as distance to group centroid, was found to be higher in the sun-exposed (harsh) habitat than in the shaded (benign) habitat (Linear model F_1,76_ = 10.1, p = 0.002, Fig 4A). This means that communities in the sun-exposed habitat were less similar among each other than the communities in the shaded habitat. However, we repeated the comparison of beta diversity between the two habitats after applying the Raup-Crick null model to the community matrix to correct for differences in alpha diversity (ANOVA, F_1,76_ = 0.1, p = 0.74, Fig 4B) and found that the significant difference in beta diversity can be explained exclusively by differences in alpha diversity.

**Fig 5:**
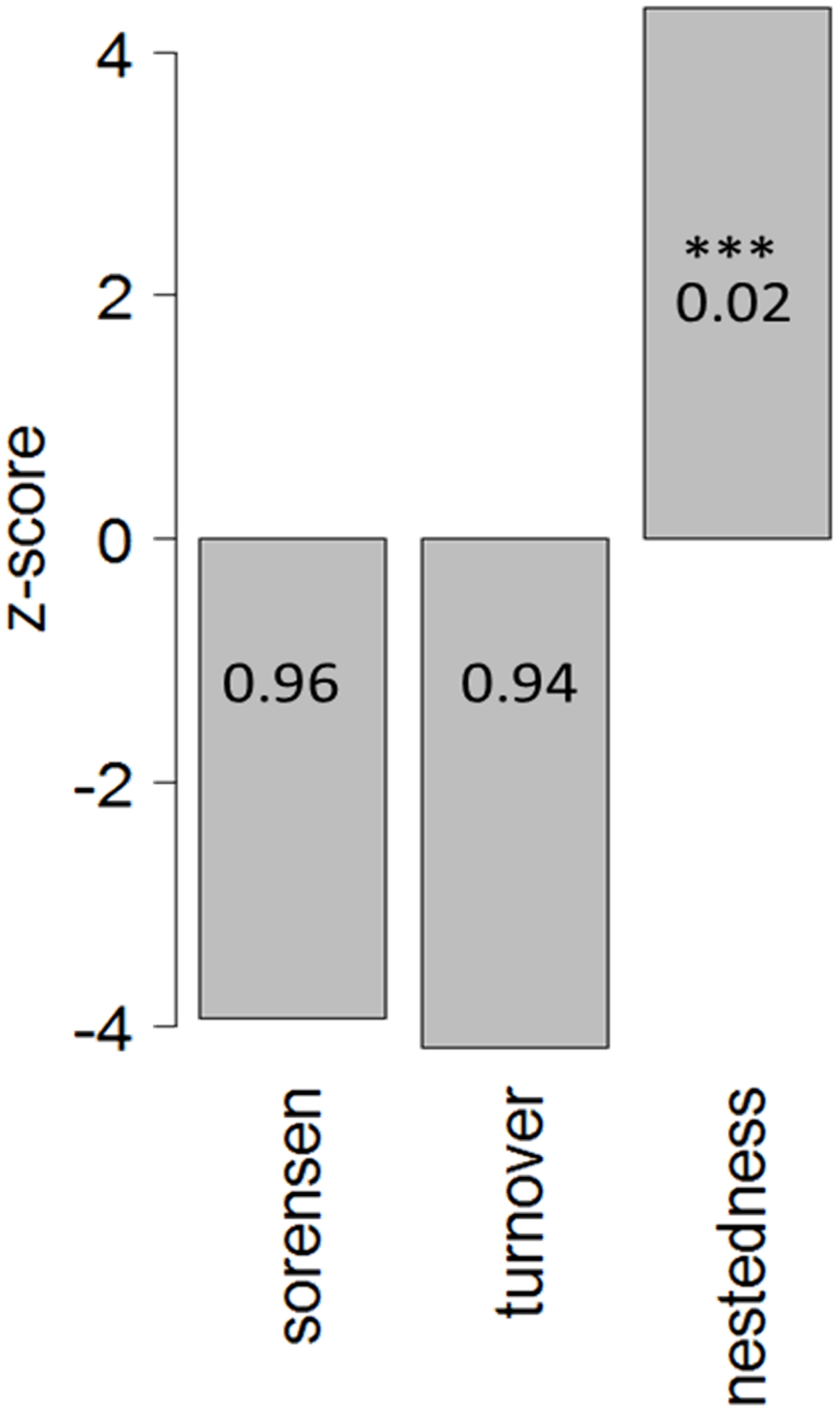
Total beta diversity (Sørensen) and partitioning into turnover and nestedness components for bromeliad microfauna communities. This analysis is based on presence-absence data. Z-scores result from 10000 simulated null model communities using the “r1”-method in the R package *vegan*. A positive z-score indicates that the value is higher than expected by chance. Whether z-scores are significantly different from zero is indicated with asterisks. ***p < 0.001. Values given with each bar show raw Sørensen, turnover and nestedness metrics. n = 78. Partitioning of beta diversity revealed that beta diversity in bromeliad microfauna communities is due to nestedness and not turnover.

To identify the mechanisms that cause potential patterns in beta diversity, we partitioned beta diversity into its two components, turnover and nestedness. A null model analysis showed that the relative importance of nestedness was significantly higher than expected (Fig 5). To further investigate if canopy cover is related to the nestedness component a nestedness rank analysis was applied. Nestedness rank showed a significantly negative relationship with canopy cover (Linear model: F_1,76_ = 10.737, p = 0.0016). This indicates that communities became less nested with an increase in canopy cover. Using alpha diversity as a co-variable in the model showed that differences in alpha diversity explained the relationship of canopy cover and nestedness rank (Linear model, alpha diversity: F_1,75_ = 388.5, p < 2*10^−16^, canopy cover fitted after alpha diversity: F_1,75_ = 2.8, p = 0.098).

## Discussion

The aim of this study was to investigate changes in bromeliad-inhabiting microfauna community composition and their community-structuring processes based on habitat characteristics along a canopy cover gradient. We found that community similarity declines with increasing environmental distance, thus supporting our first hypothesis. The amount of change in community composition, i.e. the beta diversity, differed along the canopy cover gradient, confirming our second hypothesis. The observed differences in beta diversity were linked to differences in alpha diversity. We related this finding to the change in habitat quality along the canopy cover gradient, which seems to lead to a change in the relative importance of different community assembly and maintenance processes.

### Community composition and environment

We hypothesized that an increase in environmental distance would lead to an increase in microfauna community dissimilarity along a canopy cover gradient. We found this increase in dissimilarity along the canopy cover gradient and by using null model analyses we showed that these changes were not random. We further hypothesized this change to be driven by environmental variables that change along the canopy cover gradient. Despite the fact that pH, dissolved oxygen concentration, daily fluctuations in water temperature, turbidity, mosquito larvae density and number of bromeliad leaf compartments changed along the canopy cover gradient, only daily fluctuations in water temperature, pH and number of leaves affected microfauna community composition. As the effect of the latter two parameters was only marginal (see Table 1), we focus on explaining the impact of daily fluctuations in water temperature on microfauna communities in the subsequent paragraphs. It may just be mentioned here that plant architectural complexity (in our case number of leaf compartments) can be used as a proxy for habitat heterogeneity [71] which is known to have potential effects on arthropod community composition in phytotelmata [9, 72] and that pH has been observed to affect only particular functional groups (e.g. amoeba) of bromeliad-inhabiting microfauna [36]. We suspect that habitat heterogeneity is not as important for microfauna organisms as for arthropods because microfauna organisms cannot actively rotate among the leaf compartments and we assume that the effect of pH on microfauna community is marginal because only a small part of the community is affected by it.

It has been previously observed that higher daily fluctuations in water temperature can be found in more sun-exposed bromeliads and it was suspected that this can possibly affect the survival of inhabiting taxa [73]. However, fluctuations in temperature are generally considered to be of minor importance to bromeliad-inhabiting species [74, 75]. Reasons why the importance of daily temperature fluctuations has been neglected so far could be that many former studies on bromeliad-inhabiting fauna only investigated seasonal temperature changes and not daily temperature fluctuations [76, 77]. Although we found a relatively high change in water temperature during the day for bromeliads exposed to direct sunlight (fluctuations up to 21°C), a recent work from Costa Rica suggests that local microfauna richness (i.e. alpha diversity) peaks at a relatively narrow range of temperatures (23–25°C) [36]. This indicates that the bromeliad freshwater habitat is especially challenging for thermally sensitive taxa and could explain the increase in alpha diversity with increasing canopy cover and the related decrease in temperature fluctuations. Besides, we suspect that daily water temperature fluctuations in bromeliad microcosms are even more pronounced in the dry season, when water volume is smaller due to higher evaporation and time of direct exposition to sunlight is longer due to cloudless skies. This means that effects of temperature fluctuations on microfauna communities observed in this study were potentially even more distinct if the samples would have been taken during the dry season.

In general, freshwater ecosystems—especially the smaller ones—are considered particularly vulnerable to changes in climate [78], meaning that a permanent increase in daily temperature fluctuations, e.g. by intensified weather conditions through climate change, could lead to a reduction in species richness in bromeliad micro-ecosystems due to an increase in environmental harshness as known from other studies [79–81]. A loss in species richness could have cascading effects throughout the food web and might also affect ecosystem functioning by loss of entire functional groups [82].

### Beta diversity

We hypothesized that beta diversity, i.e. the magnitude of differences in community composition, of bromeliad-inhabiting microfauna changes along the canopy cover gradient, for example due to changes in the relative importance of different community-structuring processes with habitat quality (Fig 1). Indeed, we found a change from higher beta diversity in the harsher, more sun-exposed habitat to lower beta diversity in the benign, more shaded habitat, along with a contrasting pattern in alpha diversity. The application of the Raup-Crick null model demonstrated that the pattern in beta diversity was caused by changes in alpha diversity along the habitat quality gradient. The increase of microfauna alpha diversity with an increase in habitat quality caused a distinct nestedness pattern in beta diversity. However, we could not determine the environmental variables that were responsible for the change in alpha diversity between habitats; none of our measured variables showed an effect on alpha diversity.

We found that microfauna communities from the harsher habitat were nested subsets of communities from the more benign habitat. Increasing nestedness with declining habitat quality has been previously observed for birds [83] and gastropods [84, 85]; to the best of our knowledge this is the first report of the pattern for microfauna. We found no indication that the nestedness pattern was caused by species loss from a particular microfauna group (flagellates, ciliates, amoebae, rotifers).

While alpha diversity decreased with declining habitat quality, beta diversity simultaneously increased. This increase in beta diversity in harsher environmental conditions can possibly be explained by an increase in the relative importance of stochastic processes such as demographic stochasticity and dispersal limitation [23, 27, 86].

Based on the decline in alpha diversity with increased environmental harshness we suspect that our microfauna metacommunities were subject to source-sink dynamics [43, 87] with the benign habitat providing constant immigrants of microfauna to the harsher habitat. Stochastic immigration events from the benign to the harsh environment and random extinctions, e.g. through short-time droughts or a reduction in habitat size (i.e. water volume) caused by higher temperature fluctuations, would then account for the reduced alpha diversity and higher beta diversity in the harsher habitat. Additionally, higher dispersal limitation within the harsher habitat would add to explaining the less homogeneous distribution of species and thus the higher beta diversity. Higher dispersal limitation in the harsher habitat could be partly due to lower bromeliad density compared with the more benign habitat (*personal observation*).

The higher dispersal limitation in the harsher habitat can possibly also be explained by the mode of dispersal. Microfauna organisms are passive dispersers either transported via wind or animals [88–91]. Wind dispersal requires the formation of cysts and the exposition of the cysts to wind by, for example, a complete desiccation of the bromeliad tank. However, not all microfauna species are capable of forming cysts and the bromeliads hardly ever dry up completely (*personal observation*). Therefore, the dispersal of microfauna cysts via wind is unlikely, especially over larger spatial scales [92, 93]. On the other hand, dispersal of aquatic organisms via animal agents has been commonly observed [94, 95]. In case of animal dispersal being the predominant dispersal mode, the confinement of animal activity to the more protected forested area could entail increasing dispersal limitation towards the exposed area, which would then explain its higher beta diversity. A possible increasing isolation of bromeliad tanks caused by higher dispersal limitation in the harsher, sun-exposed habitat (caused by a lower density of bromeliads and/or a lower activity of animals acting as dispersal agents) plus the lower alpha diversity in the harsher habitat are coherent with a finding by Chase and Myers [86]. They stated that isolation and low alpha diversity are accompanied by an increase in the relative importance of stochastic processes such as ecological drift, random extinctions and chance colonization. Thus, we could confirm prediction b) of our second hypothesis stating that beta diversity decreases with increasing habitat quality (Fig 1) in our system. However, Chase [26] found the opposite with environmental harshness (in his case drought) favouring the relative importance of deterministic processes (in his case strong environmental filtering) over stochastic processes. We suspect that the identity of the investigated taxa plays a major role in determining which coexistence mechanisms operate because fundamental differences can be observed in the survival strategies (e.g. active or passive dispersal) of smaller versus larger organisms [96–99]. Such taxa-dependent difference in the relative importance of coexistence mechanisms add to the complexity of conservation strategies and highlight the importance of clear conservation aims and the awareness of potential side effects for other taxa.

## Conclusion

With this study we could show that habitat characteristics describing habitat quality play an important role in structuring bromeliad-inhabiting microfauna communities, presumably through changes in the relative importance of stochastic versus deterministic processes. We observed that the extent of daily fluctuations in water temperature is a driving force of microfauna community composition and that a loss in alpha diversity with decreasing habitat quality leads to a nested pattern in beta diversity. This interlinking of alpha and beta diversity resulting in contrasting patterns in harsh versus benign habitats shows that community structure and community-structuring processes should be studied with attention to detail particularly when communities function as metacommunities. This is especially important when investigating communities with a conservation concern. So far, there is little effort in conserving microfauna [100, 101]. However, these organisms are definitely understudied, even though they provide fundamental ecosystem functions and are the base of the food web. Our analyses add to the mechanistic understanding of community dynamics in an increasingly used model system and can thus contribute to future theoretical and empirical studies.

## Supporting information

S1 Fig Relationships between the measured environmental variables in bromeliad species *Quesnelia arvensis* Mez. growing on Ilha do Cardoso, Brazil. Significant correlations (significance level = 0.05) are highlighted by colours. The colour legend indicates Pearson correlation coefficients. All measured variables are negatively related to canopy cover. CC—canopy cover [%], LC—number of leaf compartments per bromeliad, Temp—coefficient of variation of water temperature (calculated for a time frame of 23 hours), pH—pH, Turb—turbidity [NTU = nephelometric turbidity unit], DO—dissolved oxygen concentration [%], M—mosquito larva abundance [per 100 mL].
(TIF)

S1 Table Microfauna found in the bromeliad species *Quesnelia arvensis* Mez. growing in high canopy cover (shaded) environments and low canopy cover (open) environments on Ilha do Cardoso, Brazil. Morphotypes of microfauna with main morphological characteristics and their occurrence in open or shaded bromeliads are presented. H—heterotrophic nanoflagellates, C—ciliates, A—amoebae, R—rotifers. Approximate length and width are noted to give an idea about the size class and proportions.
(PDF)

## References

[1] Heywood VH, ed. Global biodiversity assessment. Cambridge: Cambridge University Press; 1995.

[2] Dudgeon D, Arthington AH, Gessner MO, Kawabata Z-I, Knowler DJ, Lévêque C et al Freshwater biodiversity: importance, threats, status and conservation challenges. Biol Rev Camb Philos Soc 2006; 81(2):163–82. doi: 10.1017/S1464793105006950 16336747 10.1017/S1464793105006950

[3] Kalinkat G, Cabral JS, Darwall W, Ficetola GF, Fisher JL, Giling DP et al Flagship umbrella species needed for the conservation of overlooked aquatic biodiversity. Conserv Biol 2017; 31(2):481–5. doi: 10.1111/cobi.12813 27558876 10.1111/cobi.12813

[4] Cowell BC, Hull HC JR., Fuller A. Recolonization of Small-Scale Disturbances by Benthic Invertebrates in Florida Freshwater Ecosystems. The Florida Entomologist 1987; 70(1):1–14.

[5] Hanazato T. Response of a zooplankton community to insecticide application in experimental ponds: a review and the implications of the effects of chemicals on the structure and functioning of freshwater communities. Environmental Pollution 1998; 101(3):361–73.

[6] Jackson DA, Peres-Neto PR, Olden JD. What controls who is where in freshwater fish communities—the roles of biotic, abiotic, and spatial factors. Can. J. Fish. Aquat. Sci. 2001; 58(1):157–70.

[7] Richardson BA, Richardson MJ, Scatena FN, Mcdowell WH. Effects of nutrient availability and other elevational changes on bromeliad populations and their invertebrate communities in a humid tropical forest in Puerto Rico. Journal of Tropical Ecology 2000; 16:167–88.

[8] Fryer G. Acidity and species diversity in freshwater crustacean faunas. Freshwater Biol 1980; 10(1):41–5.

[9] Naeem S. Resource Heterogeneity and Community Structure: A Case Study in *Heliconia imbricata* Phytotelmata. Oecologia 1990; 84(1):29–38. doi: 10.1007/BF00665591 28312771 10.1007/BF00665591

[10] Sanders RW, Wickham SA. Planktonic protozoa and metazoa: predation, food quality and population control. Marine microbial food webs 1993; 7(2):197–223.

[11] Socolar JB, Gilroy JJ, Kunin WE, Edwards DP. How Should Beta-Diversity Inform Biodiversity Conservation? Trends in Ecology & Evolution 2016; 31(1):67–80. 26701706 10.1016/j.tree.2015.11.005

[12] Al-Shami SA, Heino J, Che Salmah MR, Abu Hassan A, Suhaila AH, Madrus MR. Drivers of beta diversity of macroinvertebrate communities in tropical forest streams. Freshwater Biology 2013; 58(6):1126–37.

[13] Brendonck L, Jocqué M, Tuytens K, Timms BV, Vanschoenwinkel B. Hydrological stability drives both local and regional diversity patterns in rock pool metacommunities. Oikos 2015; 124(6):741–9.

[14] Shade A, Jones SE, McMahon KD. The influence of habitat heterogeneity on freshwater bacterial community composition and dynamics. Environ Microbiol 2008; 10(4):1057–67. doi: 10.1111/j.1462-2920.2007.01527.x 18218031 10.1111/j.1462-2920.2007.01527.x

[15] Tonkin JD, Stoll S, Jähnig SC, Haase P. Contrasting metacommunity structure and beta diversity in an aquatic-floodplain system. Oikos 2016; 125(5):686–97.

[16] Anderson MJ, Crist TO, Chase JM, Vellend M, Inouye BD, Freestone AL et al Navigating the multiple meanings of β diversity: A roadmap for the practicing ecologist. Ecology Letters 2011; 14(1):19–28. doi: 10.1111/j.1461-0248.2010.01552.x 21070562 10.1111/j.1461-0248.2010.01552.x

[17] Tuomisto H. A diversity of beta diversities: Straightening up a concept gone awry. Part 1. Defining beta diversity as a function of alpha and gamma diversity. Ecography 2010; 33(1):2–22.

[18] Tuomisto H. A diversity of beta diversities: Straightening up a concept gone awry. Part 2. Quantifying beta diversity and related phenomena. Ecography 2010; 33(1):23–45.

[19] Whittaker RH. Vegetation of the Siskiyou Mountains, Oregon and California. Ecological Monographs 1960; 30:279–338.

[20] Segre H, Ron R, de Malach N, Henkin Z, Mandel M, Kadmon R. Competitive exclusion, beta diversity, and deterministic vs. stochastic drivers of community assembly. Ecology Letters 2014; 17(11):1400–8. doi: 10.1111/ele.12343 25167950 10.1111/ele.12343

[21] Martiny JBH, Eisen JA, Penn K, Allison SD, Horner-Devine MC. Drivers of bacterial beta-diversity depend on spatial scale. Proc Natl Acad Sci U S A 2011; 108(19):7850–4. doi: 10.1073/pnas.1016308108 21518859 10.1073/pnas.1016308108 PMC3093525

[22] Wang X, Li H, Bezemer TM, Hao Z. Drivers of bacterial beta diversity in two temperate forests. Ecol Res 2016; 31(1):57–64.

[23] Arellano L, Halffter G. Gamma diversity: derived from and a determinant of Alpha diversity and Beta diversity. An analysis of three tropical landscapes. Acta Zoologica Mexicana 2003; 90:27–76.

[24] Chase JM, Biro EG, Ryberg WA, Smith KG. Predators temper the relative importance of stochastic processes in the assembly of prey metacommunities. Ecology Letters 2009; 12(11):1210–8. doi: 10.1111/j.1461-0248.2009.01362.x 19723282 10.1111/j.1461-0248.2009.01362.x

[25] Johnston NK, Pu Z, Jiang L. Predator identity influences metacommunity assembly. J Anim Ecol 2016; 85(5):1161–70. doi: 10.1111/1365-2656.12551 27349796 10.1111/1365-2656.12551

[26] Chase JM. Drought mediates the importance of stochastic community assembly. Proceedings of the National Academy of Sciences 2007; 104(44):17430–4. 10.1073/pnas.0704350104 PMC2077273 17942690

[27] Jacobsen D, Dangles O. Environmental harshness and global richness patterns in glacier-fed streams. Global Ecology and Biogeography 2012; 21(6):647–56.

[28] Peckarsky BL. Biotic interactions or abiotic limitations? A model of lotic community structure in Fontaine T. D. III and Bartell S. M., editors. Dynamics of lotic ecosystems. Ann Arbor Science, Ann Arbor Michigan, USA 1983:303–23.

[29] Blaustein L, Schwartz SS. Why study ecology in temporary pools? Israel Journal of Zoology 2001; 47(4):303–12.

[30] Srivastava DS, Kolasa J, Bengtsson J, Gonzalez A, Lawler SP, Miller TE et al Are natural microcosms useful model systems for ecology? Trends in Ecology & Evolution 2004; 19(7):379–84. 16701289 10.1016/j.tree.2004.04.010

[31] Leibold MA, Holyoak M, Mouquet N, Amarasekare P, Chase JM, Hoopes MF et al The metacommunity concept: A framework for multi-scale community ecology. Ecology Letters 2004; 7(7):601–13.

[32] Kitching RL. Food webs and container habitats The natural history and ecology of phytotelmata. Cambridge: Cambridge University Press; 2000.

[33] Williams DD. The ecology of temporary waters. Portland: Timber Press; 1987.

[34] Petermann JS, Kratina P, Marino NAC, MacDonald AAM, Srivastava DS, Salice CJ. Resources Alter the Structure and Increase Stochasticity in Bromeliad Microfauna Communities. PLoS ONE 2015; 10(3):e0118952 doi: 10.1371/journal.pone.0118952 25775464 10.1371/journal.pone.0118952 PMC4361661

[35] Srivastava DS. Habitat structure, trophic structure and ecosystem function: interactive effects in a bromeliad-insect community. Oecologia 2006; 149(3):493–504. doi: 10.1007/s00442-006-0467-3 16896779 10.1007/s00442-006-0467-3

[36] Kratina P, Petermann JS, Marino NAC, MacDonald AAM, Srivastava DS. Environmental control of the microfaunal community structure in tropical bromeliads. Ecology and evolution 2017; 7(5):1627–34. doi: 10.1002/ece3.2797 28261471 10.1002/ece3.2797 PMC5330903

[37] Rangel JV, Araújo RES, Casotti CG, Costa LC, Kiffer WP JR., Moretti MS. Assessing the role of canopy cover on the colonization of phytotelmata by aquatic invertebrates: An experiment with the tank-bromeliad Aechmea lingulata. J Limnol 2016.

[38] Serramo Lopez LC, Iglesias Rios R. Phytotelmata Faunal Communities in Sun-Exposed Versus Shaded Terrestrial Bromeliads from Southeastern Brazil. Selbyana 2001; 22(2):219–24.

[39] Farjalla VF, González AL, Céréghino R, Dézerald O, Marino NAC, Piccoli GCO et al Terrestrial support of aquatic food webs depends on light inputs: A geographically-replicated test using tank bromeliads. Ecology 2016; 97(8):2147–56. doi: 10.1002/ecy.1432 27859200 10.1002/ecy.1432

[40] de Omena PM. Effects of predators on bromeliad-aquatic arthropod communities and ecosystem functioning. Doctoral dissertation, Universidade Estadual de Campinas. 2014.

[41] Jiang L, Morin PJ. Temperature fluctuation facilitates coexistence of competing species in experimental microbial communities. J Anim Ecol 2007; 76(4):660–8. doi: 10.1111/j.1365-2656.2007.01252.x 17584371 10.1111/j.1365-2656.2007.01252.x

[42] Montagnes DJS, Weisse T. Fluctuating temperatures affect growth and production rates of planktonic ciliates. Aquatic Microbial Ecology 2000; 21:97–102.

[43] Holt RD. Spatial heterogeneity, indirect interactions, and the coexistence of prey species. The American Naturalist 1984; 124(3):377–406. 10.1086/284280 29519131

[44] Pessenda LCR, Vidotto E, de Oliveira PE, Buso AA, Cohen MCL, de Fátima Rossetti D et al Late Quaternary vegetation and coastal environmental changes at Ilha do Cardoso mangrove, southeastern Brazil. Palaeogeography, Palaeoclimatology, Palaeoecology 2012; 363:57–68.

[45] Manoel MC, Mota IZ. Análise da zona de transição da vegetação da restinga e do mangue a partir de parametros microclimáticos: estudo de caso da Ilha do Cardoso—Cananéia, SP. Revista Geonorte 2012; 3(5):734–46.

[46] Rizzini CT. Tratado de Fitogeografia do Brasil Aspectos ecológicos, sociológicos e florísticos. Rio de Janeiro: Âmbito Cultural Edições; 1997.

[47] Williams DD. The Biology of Temporary Waters. 1st ed New York: Oxford University Press; 2006.

[48] Bicca-Marques JC. Drinking Behavior in the Black Howler Monkey (Alouatta caraya). Folia Primatol 1992; 58(2):107–11. 1612535 10.1159/000156616

[49] Cestari C, Pizo MA. Utilization of epiphytes by birds in a Brazilian atlantic forest. ORNITOLOGIA NEOTROPICAL 2008; 19:97–107.

[50] Nadkarni NM, Matelson TJ. Bird Use of Epiphyte Resources in Neotropical Trees. The Condor 1989; 91(4):891–907.

[51] Dunthorn M, Stoeck T, Wolf K, Breiner H-W, Foissner W. Diversity and endemism of ciliates inhabiting Neotropical phytotelmata. Systematics and Biodiversity 2012; 10(2):195–205.

[52] Foissner W, Strüder-Kypke M, van der Staay GWM, Moon-van der Staay S-Y, Hackstein JHP. Endemic ciliates (Protozoa, Ciliophora) from tank bromeliads (Bromeliaceae): A combined morphological, molecular, and ecological study. Eur J Protistol 2003; 39(4):365–72.

[53] Schneider CA, Rasband WS, Eliceiri KW. NIH Image to ImageJ: 25 years of image analysis. Nat Meth 2012; 9(7):671–5. 10.1038/nmeth.2089 PMC5554542 22930834

[54] Faith DP, Minchin PR, Belbin L. Compositional dissimilarity as a robust measure of ecological distance In: Prentice IC, van der Maarel E, editors. Theory and models in vegetation science. Dordrecht: Springer Netherlands; 1987 p. 57–68.

[55] Oksanen J, Blanchet FG, Kindt R, Legendre P, Minchin PR, O’Hara RB et al. vegan: Community Ecology Package.: R package version 2.2–1. [http://CRAN.R-project.org/package=vegan] 2015.

[56] Poos MS, Jackson DA. Addressing the removal of rare species in multivariate bioassessments: The impact of methodological choices. Ecological Indicators 2012; 18:82–90.

[57] Legendre P, Borcard D, Peres-Neto PR. Analyzing beta diversity: Partitioning the spatial variation of community composition data. Ecological Monographs 2005; 75(4):435–50.

[58] Lichstein JW. Multiple regression on distance matrices: A multivariate spatial analysis tool. Plant Ecol 2007; 188(2):117–31.

[59] Goslee SC, Urban DL. The ecodist Package for Dissimilarity-based Analysis of Ecological Data. J. Stat. Soft. 2007; 22(7).

[60] Legendre P, Anderson MJ. Distance-Based Redundancy Analysis: Testing Multispecies Responses in Multifactorial Ecological Experiments. Ecological Monographs 1999; 69(1):1–24.

[61] Ricotta C. On beta diversity decomposition: Trouble shared is not trouble halved. Ecology 2010; 91(7):1981–3. 20715619 10.1890/09-0126.1

[62] Anderson MJ, Ellingsen KE, McArdle BH. Multivariate dispersion as a measure of beta diversity. Ecol Letters 2006; 9(6):683–93. 10.1111/j.1461-0248.2006.00926.x 16706913

[63] Chase JM, Kraft NJB, Smith KG, Vellend M, Inouye BD. Using null models to disentangle variation in community dissimilarity from variation in α-diversity. Ecosphere 2011; 2(2):art24.

[64] Baselga A, Orme D, Villeger S, Bortoli J de, Leprieur F. betapart: Partitioning beta diversity into turnover and nestedness components.: R package version 1.3. [http://CRAN.R-project.org/package=betapart] 2013.

[65] Baselga A. Partitioning the turnover and nestedness components of beta diversity. Global Ecology and Biogeography 2010; 19(1):134–43.

[66] Patterson BD, Atmar W. Nested subsets and the structure of insular mammalian faunas and archipelagos. Biological Journal of the Linnean Society 1986; 28:65–82.

[67] Almeida-Neto M, Guimarães P, Guimarães PR JR., Loyola RD, Ulrich W. A consistent metric for nestedness analysis in ecological systems: reconciling concept and measurement. Oikos 2008; 117:1227–39.

[68] Ulrich W. Nestedness analysis as a tool to identify ecological gradients. Ecological Questions 2009; 11:27–34.

[69] Dormann CF, Gruber B, Fründ J. Introducing the bipartite Package:Analysing Ecological Networks. R News 2008; 8(2):8–11.

[70] R Core Team. R: A language and environment for statistical computing; 2013 URL: URL http://www.R-project.org/.

[71] Tews J, Brose U, Grimm V, Tielbörger K, Wichmann MC, Schwager M et al Animal species diversity driven by habitat heterogeneity/diversity: the importance of keystone structures. Journal of Biogeography 2004; 31(1):79–92.

[72] Gonçalves-Souza T, Almeida-Neto M, Romero GQ. Bromeliad architectural complexity and vertical distribution predict spider abundance and richness. Austral Ecology 2011; 36(4):476–84.

[73] Laessle AM. A Micro-Limnological Study of Jamaican Bromeliads. Ecology 1961; 42(3):499–517.

[74] Weisse T, Scheffel U, Stadler P, Foissner W. Bromeliothrix metopoides, a boom and bust ciliate (Ciliophora, Colpodea) from tank bromeliads. Eur J Protistol 2013; 49(3):406–19. doi: 10.1016/j.ejop.2013.02.001 23541138 10.1016/j.ejop.2013.02.001 PMC3688101

[75] Weisse T, Scheffel U, Stadler P, Foissner W. Functional ecology of the ciliate Glaucomides bromelicola, and comparison with the sympatric species Bromeliothrix metopoides. J Eukaryot Microbiol 2013; 60(6):578–87. doi: 10.1111/jeu.12063 23865693 10.1111/jeu.12063 PMC4028988

[76] Marino NAC, Guariento RD, Dib V, Azevedo FD, Farjalla VF. Habitat size determine algae biomass in tank-bromeliads. Hydrobiologia 2011; 678(1):191–9.

[77] Mestre LAM, Aranha JMR, Esper MLP. Macroinvertebrate Fauna Associated to the Bromeliad Vriesea inflata of the Atlantic Forest (Paraná State, Southern Brazil). Brazilian Archives of Biology and Technology 2001; 44(1):89–94.

[78] Woodward G, Perkins DM, Brown LE. Climate change and freshwater ecosystems: impacts across multiple levels of organization. Philos Trans R Soc Lond B Biol Sci 2010; 365(1549):2093–106. doi: 10.1098/rstb.2010.0055 20513717 10.1098/rstb.2010.0055 PMC2880135

[79] Marks CO, Muller-Landau HC, Tilman D. Tree diversity, tree height and environmental harshness in eastern and western North America. Ecology Letters 2016; 19(7):743–51. doi: 10.1111/ele.12608 27146846 10.1111/ele.12608

[80] Staddon WJ, Trevors JT, Duchesne LC, Colombo CA. Soil microbial diversity and community structure across a climatic gradient in western Canada. Biodiversity and Conservation 1998; 7:1081–92.

[81] Whittaker RJ, Willis KJ, Field R. Scale and species richness: towards a general, hierarchical theory of species diversity. Journal of Biogeography 2001; 28:453–70.

[82] Petchey OL, McPhearson PT, Casey TM, Morin PJ. Environmental warming alters food-web structure and ecosystem function. Nature 1999; 402(6757):69–72.

[83] Fernández-Juricic E. Can human disturbance promote nestedness?: A case study with breeding birds in urban habitat fragments. Oecologia 2002; 131(2):269–78. doi: 10.1007/s00442-002-0883-y 28547695 10.1007/s00442-002-0883-y

[84] Bloch CP, Higgins CL, Willig MR. Effects of Large-Scale Disturbance on Metacommunity Structure of Terrestrial Gastropods: Temporal Trends in Nestedness. Oikos 2007; 116(3):395–406.

[85] Hylander K, Nilsson C, Jonsson BG, Göthner T. Differences in Habitat Quality Explain Nestedness in a Land Snail Meta-Community. Oikos 2005; 108(2):351–61.

[86] Chase JM, Myers JA. Disentangling the importance of ecological niches from stochastic processes across scales. Philosophical Transactions of the Royal Society B: Biological Sciences 2011; 366(1576):2351–63. 10.1098/rstb.2011.0063 PMC3130433 21768151

[87] Pulliam HR. Sources, Sinks, and Population Regulation. The American Naturalist 1988; 132(5):652–61.

[88] Maguire B JR. The Passive Dispersal of Small Aquatic Organisms and Their Colonization of Isolated Bodies of Water. Ecological Monographs 1963; 33(2):161–85.

[89] Revill DL, Stewart KW, Schlichting HE JR. Passive Dispersal of Viable Algae and Protozoa By Certain Craneflies and Midges. Ecology 1967; 48(6):1023–7.

[90] Rogerson A, Detwiler A. Abundance of airborne heterotrophic protists in ground level air of South Dakota. Atmospheric Research 1999; 51(1):35–44.

[91] Schlichting HE JR., Sides SL. The Passive Transport of Aquatic Microorganisms by Selected Hemiptera. Journal of Ecology 1969; 57(3):759–64.

[92] Foissner W. Biogeography and Dispersal of Micro-organisms: A Review Emphasizing Protists. Acta Protozoologica 2006; 45:111–36.

[93] Horváth Z, Vad CF, Ptacnik R. Wind dispersal results in a gradient of dispersal limitation and environmental match among discrete aquatic habitats. Ecography 2016; 39(8):726–32. doi: 10.1111/ecog.01685 28529408 10.1111/ecog.01685 PMC5438046

[94] Serramo Lopez LC, Rodrigues Pena, Pablo J. F., Iglesias Rios R. Frogs and Snakes as Phoretic Dispersal Agents of Bromeliad Ostracods (Limnocytheridae: Elpidium) and Annelids (Naididae: Dero). Biotropica 1999; 31(4):705–8.

[95] Vanschoenwinkel B, Waterkeyn A, Nhiwatiwa T, Pinceel TOM, Spooren E, Geerts A et al Passive external transport of freshwater invertebrates by elephant and other mud-wallowing mammals in an African savannah habitat. Freshwater Biology 2011; 56(8):1606–19.

[96] Astorga A, Oksanen J, Luoto M, Soininen J, Virtanen R, Muotka T. Distance decay of similarity in freshwater communities: Do macro- and microorganisms follow the same rules? Global Ecology and Biogeography 2012; 21(3):365–75.

[97] Farjalla VF, Srivastava DS, Marino NAC, Azevedo FD, Dib V, Lopes PM et al Ecological determinism increases with organism size. Ecology 2012; 93(7):1752–9. 22919920 10.1890/11-1144.1

[98] Finlay B, Esteban GF, Fenchel T. Protist Diversity is Different? Protist 2004; 155(1):15–22. 15144054 10.1078/1434461000160

[99] Nemergut DR, Schmidt SK, Fukami T, O’Neill SP, Bilinski TM, Stanish LF et al Patterns and processes of microbial community assembly. Microbiol Mol Biol Rev 2013; 77(3):342–56. doi: 10.1128/MMBR.00051-12 24006468 10.1128/MMBR.00051-12 PMC3811611

[100] Cotterill FPD, Al-Rasheid K, Foissner W. Conservation of protists: Is it needed at all? Biodivers Conserv 2008; 17(2):427–43.

[101] Esteban GF, Finlay BJ. Conservation work is incomplete without cryptic biodiversity. Nature 2010; 463(7279):293. 10.1038/463293c 20090730

