# Supplementary figures and images for: Different in the dark: The effect of habitat characteristics on community composition and beta diversity in bromeliad microfauna
Source: PLoS One. 2018 Feb 5;13(2):e0191426. doi: 10.1371/journal.pone.0191426 (PMC5798767; doi:10.1371/journal.pone.0191426)

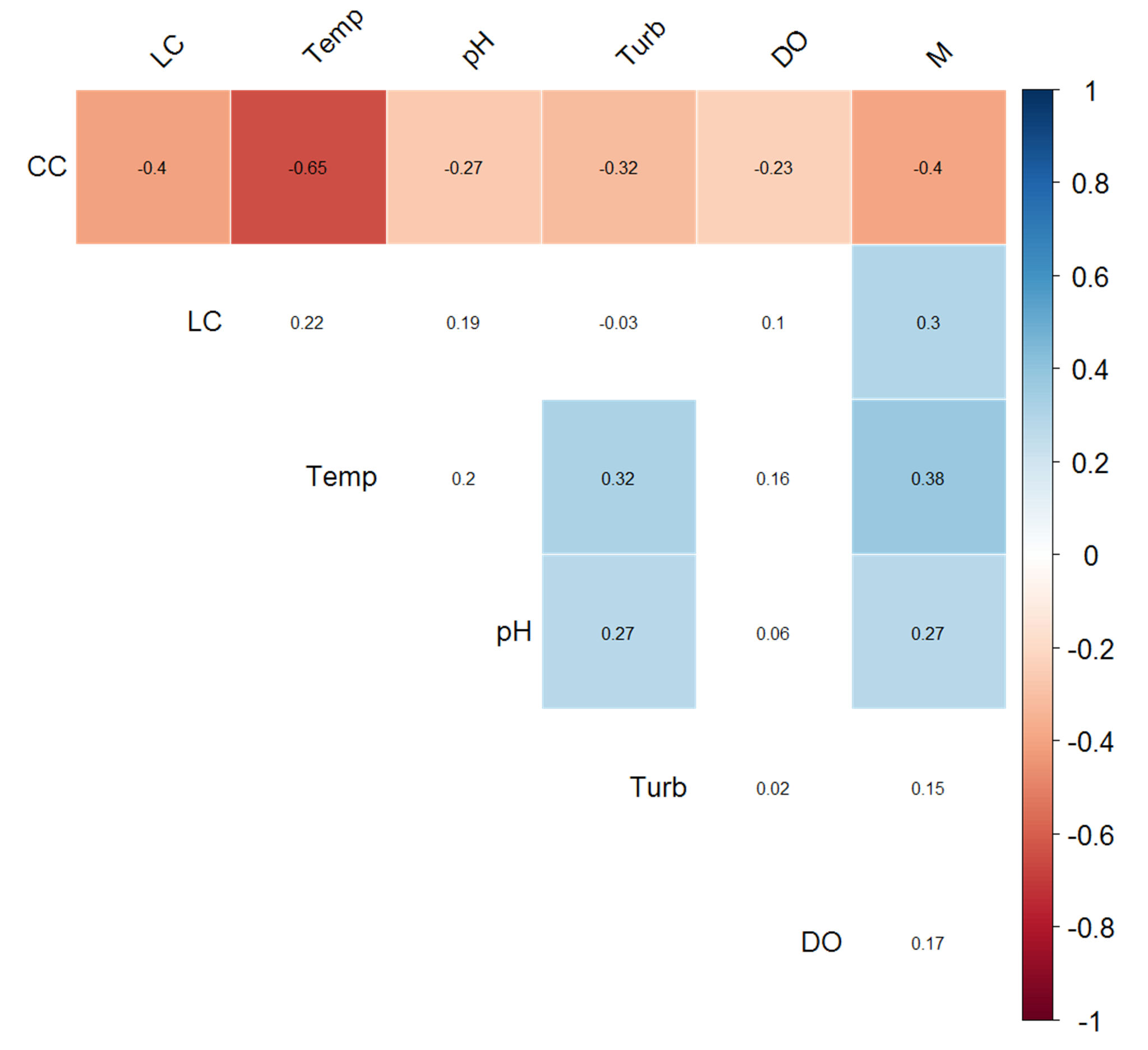

Supplement: S1 Fig — Significant correlations (significance level = 0.05) are highlighted by colours. The colour legend indicates Pearson correlation coefficients. All measured variables are negatively related to canopy cover. CC—canopy cover [%], LC—number of leaf compartments per bromeliad, Temp—coefficient of variation of water temperature (calculated for a time frame of 23 hours), pH—pH, Turb—turbidity [NTU = nephelometric turbidity unit], DO—dissolved oxygen concentration [%], M—mosquito larva abundance [per 100 mL]. (TIF) [file pone.0191426.s001.tif]
