# Supplementary material for: Different in the dark: The effect of habitat characteristics on community composition and beta diversity in bromeliad microfauna
Source: PLoS One. 2018 Feb 5;13(2):e0191426. doi: 10.1371/journal.pone.0191426 (PMC5798767; doi:10.1371/journal.pone.0191426)
Supplement: S1 Table — Morphotypes of microfauna with main morphological characteristics and their occurrence in open or shaded bromeliads are presented. H—heterotrophic nanoflagellates, C—ciliates, A—amoebae, R—rotifers. Approximate length and width are noted to give an idea about the size class and proportions. (PDF) [file pone.0191426.s002.pdf]

**S1 Table:** Microfauna found in the bromeliad species *Quesnelia arvensis* Mez. growing in high canopy cover (shaded) environments and low canopy cover (open) environments on Ilha do Cardoso, Brazil. Morphotypes of microfauna with main morphological characteristics and their occurrence in open or shaded bromeliads are presented. H – heterotrophic nanoflagellates, C – ciliates, A – amoebae, R – rotifers. Approximate length and width are noted to give an idea about the size class and proportions.

| **Name** | **≈ Length x width [µm]** | **Morphological characteristics** | **Occurrence** | |
|---|---|---|---|---|
| | | | **Open** | **Shaded** |
| H1 | 20 x 5 | Lanceolated shape, one long flagellum | x | x |
| H2 | 5 x 5 | Ovoid shape, two short flagella | x | x |
| H3 | 5 x 5 | Circular shape, one short flagellum | x | x |
| H4 | 6 x 7 | Round cell, two flagella (one short, one long) | x | x |
| H5 | 7 x 7 | Ovoid shape, two long flagella | x | x |
| H6 | 15 x 5 | Bean-shaped, one flagellum | x | x |
| H7 | 10 x 4 | Lanceolated shape, four flagella | x | x |
| H8 | 10 x 5 | Teardrop-shaped, two long flagella | x | x |
| H9 | 10 x 5 | Ellipsoid to square shaped, two short flagella | | x |
| H10 | 15 x 5 | Oval shape, two flagella (one short, one long) | | x |
| H11 | 40 x 8 | Elongated ellipsoid shape, one long flagellum | x | |
| H12 | 8 x 7 | Round shape, one long flagellum | x | x |
| H13 | 15 x 5 | Drop-shaped, one flagellum | x | x |
| H14 | 20 x 4 | Elongated-ovoid shape, one flagellum | x | x |
| H15 | 5 x 5 | Round cell, two long flagella | x | x |
| C1 | 30 x 20 | Kidney-shaped, entirely covered with cilia | x | x |
| C2 | 15 x 7 | Oval shaped, entirely covered with cilia | x | x |
| C3 | 15 x 7 | Ellipsoid shape, few long cilia located on both ends | x | x |
| C4 | 35 x 15 | Ovoid shape,<br>entirely covered with cilia | x | x |
| C5 | 10 x 5 | Ellipsoid to square shape, entirely covered with cilia | x | x |
| C6 | 15 x 10 | Pear-shaped, cilia at one end of the body | x | |
| C7 | 15 x 15 | Circular to oval shape, long cilia around entire body | x | x |
| C8 | 7 x 7 | Round shape, short cilia | x | x |
| C9 | 50 x 15 | Lanceolated shape, mouth cavity | x | x |
| A1 | 15 x 15 | Naked amoeba, shapeless cell, with pseudopodia | x | x |
| A2 | 30 x 15 | Vase-like shape, round mouth part on side | x | x |
| A3 | 30 x 15 | Oval shape, aperture with teeth | x | x |
| A4 | 65 x 35 | Hemispherical elongated shape, aperture with teeth | | x |
| R1 | 90 x 50 | Loricate, one toe, head aperture with concave margins | x | x |
| R2 | 60 x30 | Loricate, two toes, head aperture with concave margins of different size | | x |
| R3 | 70 x 40 | Iloricate, elongated foot | x | x |
| R4 | 80 x 60 | Loricate, head aperture margins straight, one toe | x | x |
| R5 | 120 x 60 | Iloricate, elongated foot | x | x |
| R6 | 50 x 40 | Iloricate, small | x | x |
| R7 | 100 x 50 | Loricate, lorica outline nearly circular, two long toes | x | x |
